# Supplementary material for: SuperCellCyto: enabling efficient analysis of large scale cytometry datasets
Source: Genome Biol. 2024 Apr 8;25:89. doi: 10.1186/s13059-024-03229-3 (PMC11003185; doi:10.1186/s13059-024-03229-3)
Supplement: Supplementary file 1 — Additional file 1. SuperCellCyto: enabling efficient analysis of large scale cytometry datasets. Supplementary file containing Note S1, Table S1-S7, and Fig. S1-S19. [file 13059_2024_3229_MOESM1_ESM.docx]

SuperCellCyto: enabling efficient analysis of large scale cytometry datasets Additional File 1

Givanna H. Putri^1,*^, George Howitt^2^, Felix Marsh-Wakefield^3^, Thomas M. Ashhurst^4^, Belinda Phipson^1,*^

1 The Walter and Eliza Hall Institute of Medical Research and The Department of Medical Biology, The University of Melbourne, Parkville, VIC, Australia

2 Peter MacCallum Cancer Centre and The Sir Peter MacCallum Department of Oncology, The University of Melbourne, Parkville, VIC, Australia

3 Centenary Institute of Cancer Medicine and Cell Biology, The University of Sydney Sydney, NSW, Australia

4 Sydney Cytometry Core Research Facility and School of Medical Sciences, The University of Sydney, Sydney, NSW, Australia

* Correspondence to: GHP: [putri.g@wehi.edu.au](mailto:putri.g@wehi.edu.au), BP: [phipson.b@wehi.edu.au](mailto:phipson.b@wehi.edu.au)

#

#

# Note S1

The manual gating strategy employed by Oetjen et al. for the identification of B cell subsets, reproduced from the original manuscript [45]:

- Pre-B cell (CD20-): CD45+ CD19+ CD20- CD10+
- Immature B cells (CD20+): CD45+ CD19+ CD20+ CD10+
  - Transitional B cells: CD45+ CD19+ CD20+ CD10+ CD27-
- Mature B cells: CD45+ CD19+ CD20+ CD10-
- Naïve Mature B cells: CD45+ CD19+ CD20+ CD10- CD27- CD21hi
- Exhausted/Tissue-like Memory: CD45+ CD19+ CD20+ CD10- CD27- CD21lo
- Memory B cells: CD45+ CD19+ CD20+ CD10- CD27+
- Activated Mature: CD45+ CD19+ CD20+ CD10- CD27+ CD21lo
- Resting Memory: CD45+ CD19+ CD20+ CD10- CD27+ CD21hi
- CD20- CD10-: CD45+ CD19+ CD20- CD10-
- Plasmablast: CD45+ CD19+ CD20- CD10- CD27+ CD38+
- Plasma Cell: CD45+ CD19+ CD20- CD10- CD27+ CD38+ CD138+

| Dataset | No. of markers | No. of cells | No. of samples | Tissue | Species | Reference |
| --- | --- | --- | --- | --- | --- | --- |
| Levine_32dim | 32 | 265,627 | 2 | Bone Marrow | Human | [7] |
| Samusik_all | 39 | 841,644 | 10 | Bone Marrow | Mouse | [[](https://www.zotero.org/google-docs/?V0GEcZ)8] |
| Oetjen_bcells | 13 | 8,314,260 | 22 | Bone Marrow | Human | [45] |
| Trussart_cytofruv | 31 | 8,589,739 | 24 (12 independent) | PBMC | Human | [46] |
| BCR_XL | 24 | 172,791 | 16 (8 independent) | PBMC | Human | [49] |
| Anti_PD1 | 24 | 85,715 | 20 | PBMC | Human | [50] |
| CITEseq | 97 | 49,057 | 10 | Bone Marrow | Human | [52] |

Table S1. Overview of datasets used to verify the effectiveness of supercells. All datasets have been previously published, and references to these publications can be found in the final column.

| Dataset | Number of cells | Gamma | Number of supercells |
| --- | --- | --- | --- |
| Levine_32dim | 265,627 | 5 | 53,125 |
|  |  | 10 | 26,563 |
|  |  | 15 | 17,709 |
|  |  | 20 | 13,282 |
|  |  | 25 | 10,625 |
|  |  | 30 | 8,854 |
|  |  | 35 | 7,589 |
|  |  | 40 | 6,641 |
|  |  | 45 | 5,903 |
|  |  | 50 | 5,313 |
| Samusik_all | 841,644 | 5 | 168,328 |
|  |  | 10 | 84,164 |
|  |  | 15 | 56,109 |
|  |  | 20 | 42,082 |
|  |  | 25 | 33,666 |
|  |  | 30 | 28,055 |
|  |  | 35 | 24,048 |
|  |  | 40 | 21,042 |
|  |  | 45 | 18,702 |
|  |  | 50 | 16,832 |

Table S2. The number of supercells generated by various gamma values for Levine_32dim [7] and Samusik_all [8] datasets.

**A**

| **Number of metaclusters** | **Grid size** |
| --- | --- |
| 15 | 10 |
| 20 | 10 |
| 25 | 10 |
| 30 | 10 |
| 15 | 11 |
| 20 | 11 |
| 25 | 11 |
| 30 | 11 |
| 15 | 12 |
| 20 | 12 |
| 25 | 12 |
| 30 | 12 |
| 15 | 13 |
| 20 | 13 |
| 25 | 13 |
| 30 | 13 |
| 15 | 14 |
| 20 | 14 |
| 25 | 14 |
| 30 | 14 |

**B**

| **k** |
| --- |
| 10 |
| 15 |
| 20 |
| 25 |
| 30 |

Table S3. Range of parameter values explored for clustering Levine_32dim and Samusik_all datasets using (A) FlowSOM [[6]](https://www.zotero.org/google-docs/?0gGA5R) and (B) Louvain [[44]](https://www.zotero.org/google-docs/?Zuajgq) algorithms. For FlowSOM, a square grid was employed for the Self Organising Map (SOM). The grid size represents the size of the square grid. For example, a grid size of 10 represents a square SOM grid of size 10x10.

**A**

| **Gamma** | **< 0.5** | **0.5 (inc.) - 0.9 (exc.)** | **0.9 (inc.) - 1 (exc.)** | **1** |
| --- | --- | --- | --- | --- |
| 5 | 1 (0.002%) | 0 (0.0%) | 1,546 (3.23%) | 46,376 (96.77%) |
| 10 | 5 (0.02%) | 1,635 (6.31%) | 12 (0.05%) | 24,248 (93.62%) |
| 15 | 7 (0.04%) | 1,328 (7.59%) | 94 (0.54%) | 16,070 (91.83%) |
| 20 | 5 (0.04%) | 1,076 (8.17%) | 152 (1.15%) | 11,941 (90.64%) |
| 25 | 8 (0.08%) | 901 (8.54%) | 178 (1.69%) | 9,462 (89.7%) |
| 30 | 7 (0.08%) | 776 (8.83%) | 196 (2.23%) | 7,811 (88.86%) |
| 35 | 9 (0.12%) | 665 (8.82%) | 202 (2.68%) | 6,663 (88.38%) |
| 40 | 8 (0.12%) | 587 (8.9%) | 207 (3.14%) | 5,797 (87.85%) |
| 45 | 3 (0.05%) | 525 (8.95%) | 197 (3.36%) | 5,140 (87.64%) |
| 50 | 3 (0.06%) | 483 (9.15%) | 193 (3.66%) | 4,599 (87.14%) |

**B**

| **Gamma** | **< 0.5** | **0.5 (inc) - 0.9 (exc.)** | **0.9 (inc.) - 1 (exc.)** | **1** |
| --- | --- | --- | --- | --- |
| 5 | 146 (0.1%) | 11,170 (7.98%) | 51 (0.04%) | 128,599 (91.88%) |
| 10 | 258 (0.35%) | 10,573 (14.28%) | 1,253 (1.69%) | 61,979 (83.68%) |
| 15 | 299 (0.59%) | 8,610 (17.12%) | 2,231 (4.44%) | 39,151 (77.85%) |
| 20 | 305 (0.8%) | 7,211 (18.93%) | 2,579 (6.77%) | 27,999 (73.5%) |
| 25 | 278 (0.91%) | 6,255 (20.42%) | 2,665 (8.7%) | 21,430 (69.97%) |
| 30 | 281 (1.1%) | 5,554 (21.67%) | 2,626 (10.24%) | 17,174 (66.99%) |
| 35 | 268 (1.22%) | 5,027 (22.8%) | 2,628 (11.92%) | 14,127 (64.07%) |
| 40 | 251 (1.3%) | 4,586 (23.71%) | 2,568 (13.28%) | 11,936 (61.71%) |
| 45 | 247 (1.43%) | 4,222 (24.51%) | 2,482 (14.41%) | 10,275 (59.65%) |
| 50 | 239 (1.54%) | 3,898 (25.1%) | 2,417 (15.57%) | 8,973 (57.79%) |

Table S4. Breakdown of the purity score obtained for the (A) Levine_32dim [[7]](https://www.zotero.org/google-docs/?QAnYE2) dataset and (B) Samusik_all [[8]](https://www.zotero.org/google-docs/?T059cV) dataset across all gamma values. *Inc.* stands for inclusive, while *exc.* represents exclusive.

| **Cytometry Data Cell Type** | **CITEseq Data Cell Type** |
| --- | --- |
| CD16-_NK_cells | CD56brightCD16- NK cells |
| CD16+_NK_cells | CD56dimCD16+ NK cells |
| CD4_T_cells | CD4+ cytotoxic T cells,  CD4+ memory T cells,  Naive CD4+T cells |
| CD8_T_cells | CD8+ central memory T cells,  CD8+ effector memory T cells,  CD8+ naive T cells,  CD8+CD103+ tissue resident memory T cells |
| Mature_B_cells | CD11c+ memory B cells,  Mature naive B cells,  Class switched memory B cells,  Nonswitched memory B cells |
| Monocytes | Classical Monocytes,  Non-classical monocytes |
| pDCs | Plasmacytoid dendritic cells |
| Plasma_B_cells | Plasma cells |
| Pre_B_cells | Small pre-B cell |
| Pro_B_cells | pro-B cells |
| CD34+_HSCs_and_HSPCs | Lymphoid-primed multipotent progenitors,  Megakaryocyte progenitors,  Erythro-myeloid progenitors,  NK cell progenitors,  HSCs & MPPs |

Table S5. Mapping of cell type labels between Levine_32dim [[7]](https://www.zotero.org/google-docs/?wtx7Qd) cytometry data and the CITEseq data [[52]](https://www.zotero.org/google-docs/?dPSwy9).

| **Cell type** | **Proportion** |
| --- | --- |
| CD4_T_cells | 0.25307149 |
| Monocytes | 0.2025167 |
| CD8_T_cells | 0.19300468 |
| Mature_B_cells | 0.15856561 |
| Pre_B_cells | 0.0588862 |
| CD34+_HSCs_and_HSPCs | 0.04333679 |
| CD16-_NK_cells | 0.03748176 |
| CD16+_NK_cells | 0.02157721 |
| pDCs | 0.01188282 |
| Basophils | 0.01158527 |
| Pro_B_cells | 0.00492398 |
| Plasma_B_cells | 0.00316747 |

Table S6. Proportions of cell types in Levine_32dim cytometry data [[7]](https://www.zotero.org/google-docs/?THjoCt). Each row corresponds to a specific cell type, with the proportion of cells belonging to that cell.

| **Process** | **Algorithm** | **Resolution** | **Dataset** | **Platform Used** |
| --- | --- | --- | --- | --- |
| Supercells Generation | SuperCellCyto | Single Cells | Anti_PD1,  BCR_XL,  Levine_32dim,  Oetjen_bcells,  Samusik_all,  Trussart_cytofruv | 2022 MacBook Pro (M2 chip, 24GB RAM) |
| Clustering | FlowSOM | Supercells,  Single Cells | Levine_32dim,  Samusik_all | 2022 MacBook Pro (M2 chip, 24GB RAM) |
| Clustering | Louvain | Supercells | Levine_32dim,  Samusik_all | 2022 MacBook Pro (M2 chip, 24GB RAM) |
| Batch Correction | cyCombine | Supercells,  Single Cells | Trussart_cytofruv | 2022 MacBook Pro (M2 chip, 24GB RAM) |
| Clustering | Louvain | Supercells,  Single Cells | Oetjen_bcells | High-Performance Computing (HPC) Platform |
| Clustering | Louvain | Single Cells | Levine_32dim,  Samusik_all | High-Performance Computing (HPC) Platform |
| Batch Correction | CytofRUV | Supercells,  Single Cells | Trussart_cytofruv | High-Performance Computing (HPC) Platform |

Table S7. The computing platforms used for benchmarking the performance of various analysis processes. Nextflow [[53]](https://www.zotero.org/google-docs/?z5sF7f) pipelines were used to run the processes on the High Performance Computing (HPC) platform. The amount of RAM and CPUs allocated are specified within the Nextflow scripts available on <https://phipsonlab.github.io/SuperCellCyto-analysis/>.


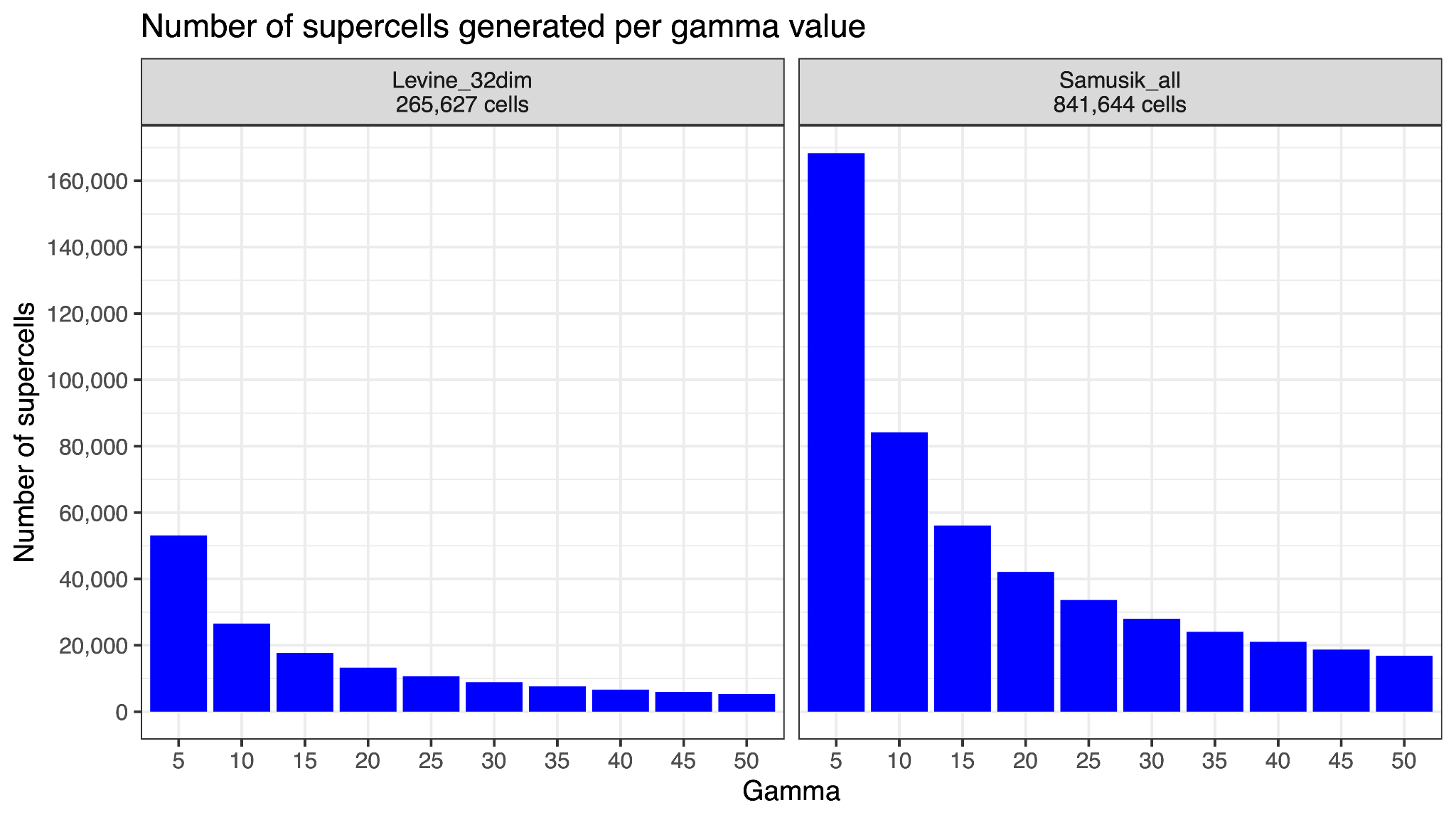


Fig. S1. Number of supercells generated for Levine_32dim [[7]](https://www.zotero.org/google-docs/?wibLef) and Samusik_all [[8]](https://www.zotero.org/google-docs/?tb1uNJ) datasets across various gamma values.


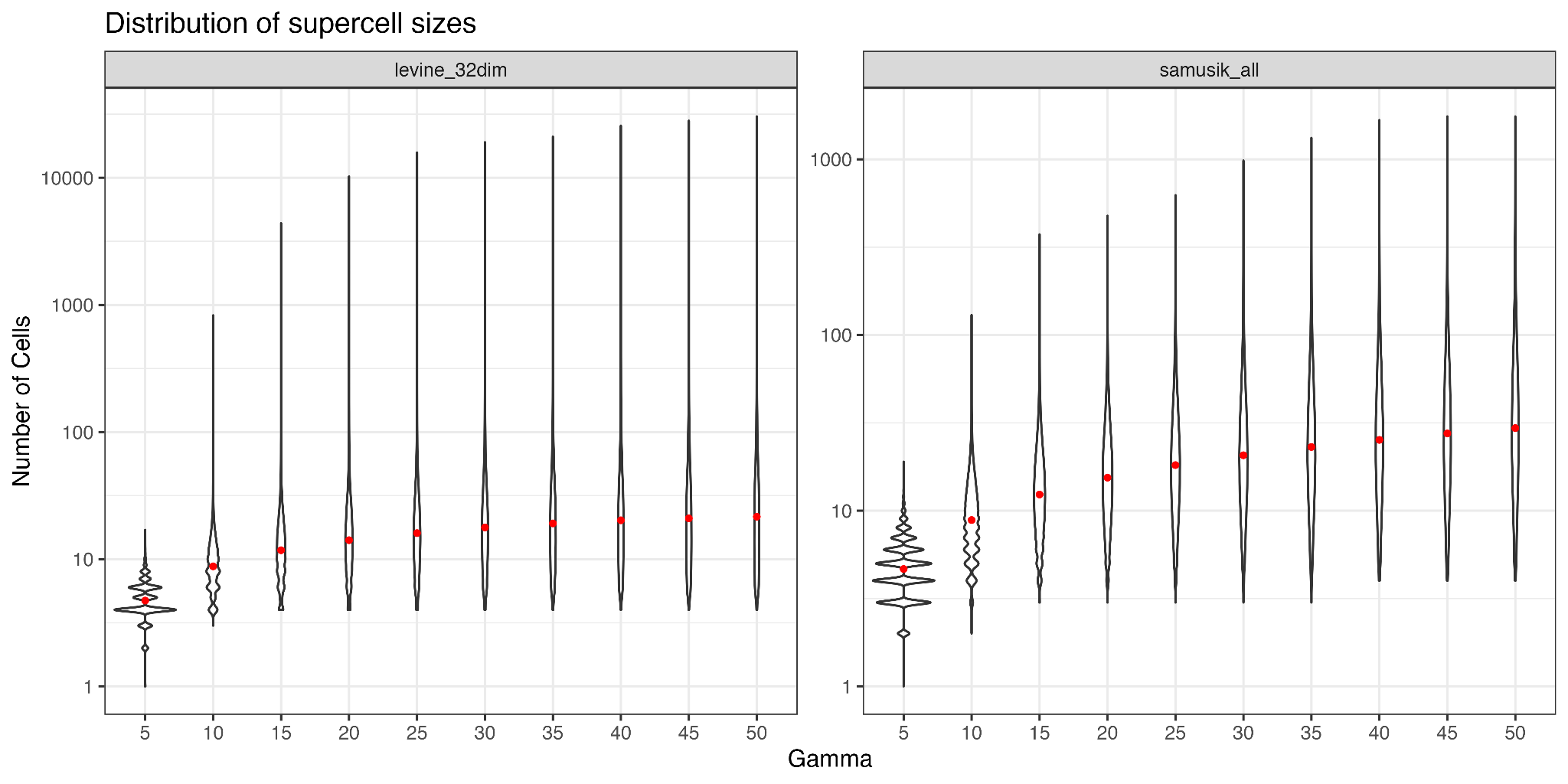


Fig. S2. Distribution of the number of cells captured in the supercells generated for Levine_32dim [[7]](https://www.zotero.org/google-docs/?q42Vvj) and Samusik_all [[8]](https://www.zotero.org/google-docs/?urEWuZ) datasets using different gamma values. The red dot denotes the mean of the distribution.


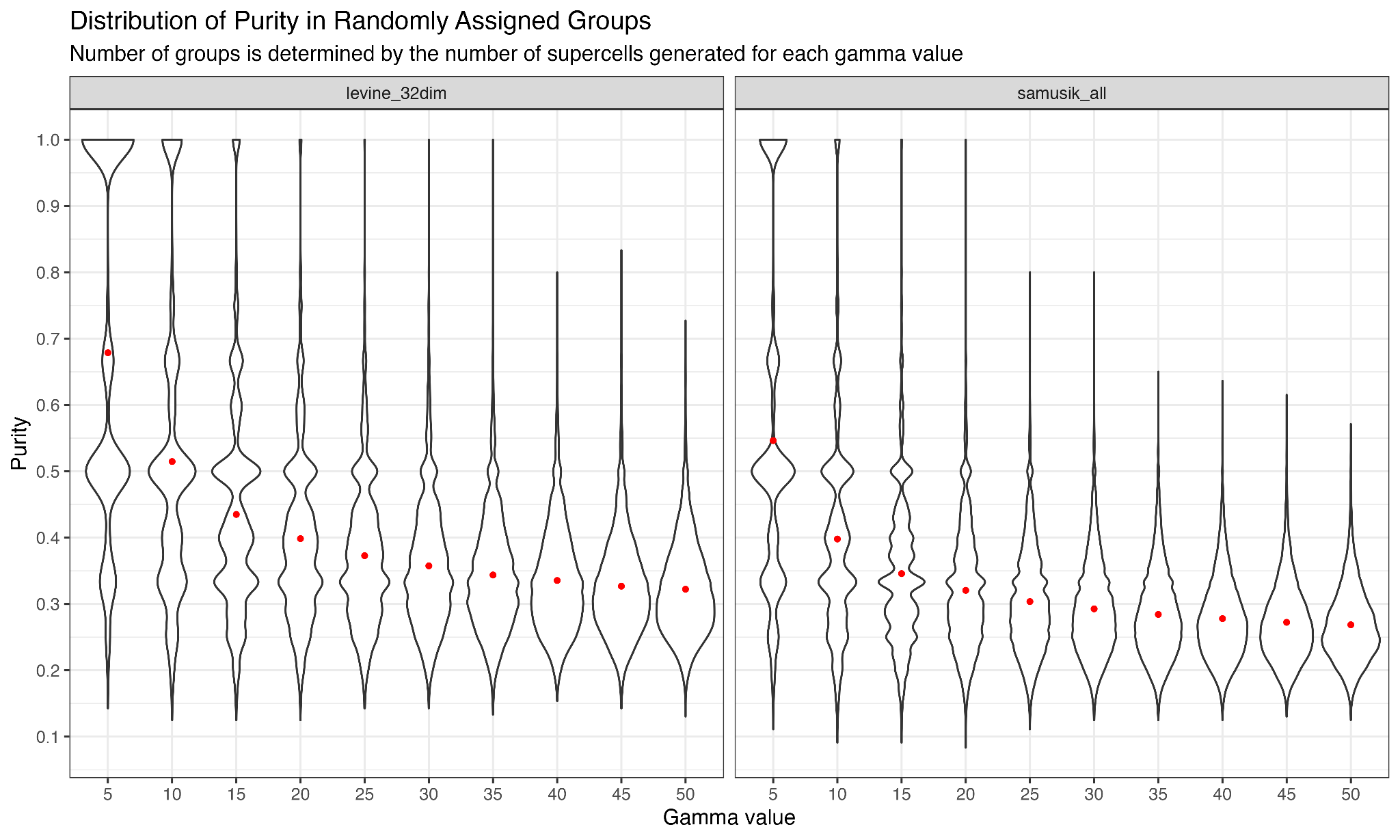


Fig. S3. Violin plot illustrating the purity score of cell groupings, where cells are randomly assigned into groups. The number of groups corresponds to the number of supercells generated for specific gamma values for a given dataset. See Additional file 1: Fig. S1 for the number of supercells generated for a given dataset and gamma value.


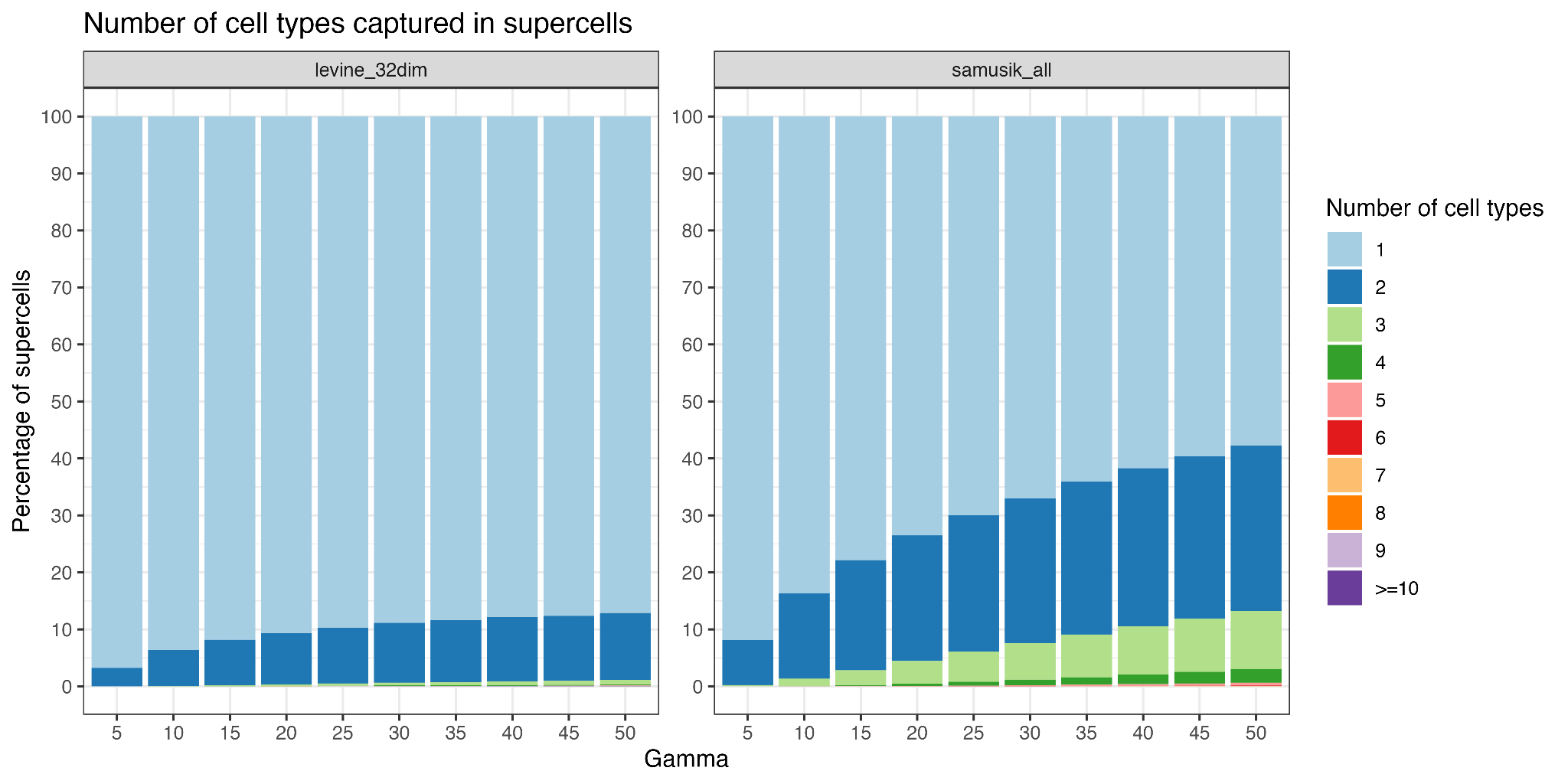


Fig. S4. Number of different cell types captured in supercells generated for Levine_32dim [[7]](https://www.zotero.org/google-docs/?5vvEJS) and Samusik_all [[8]](https://www.zotero.org/google-docs/?6BUnB4) datasets using different gamma values.


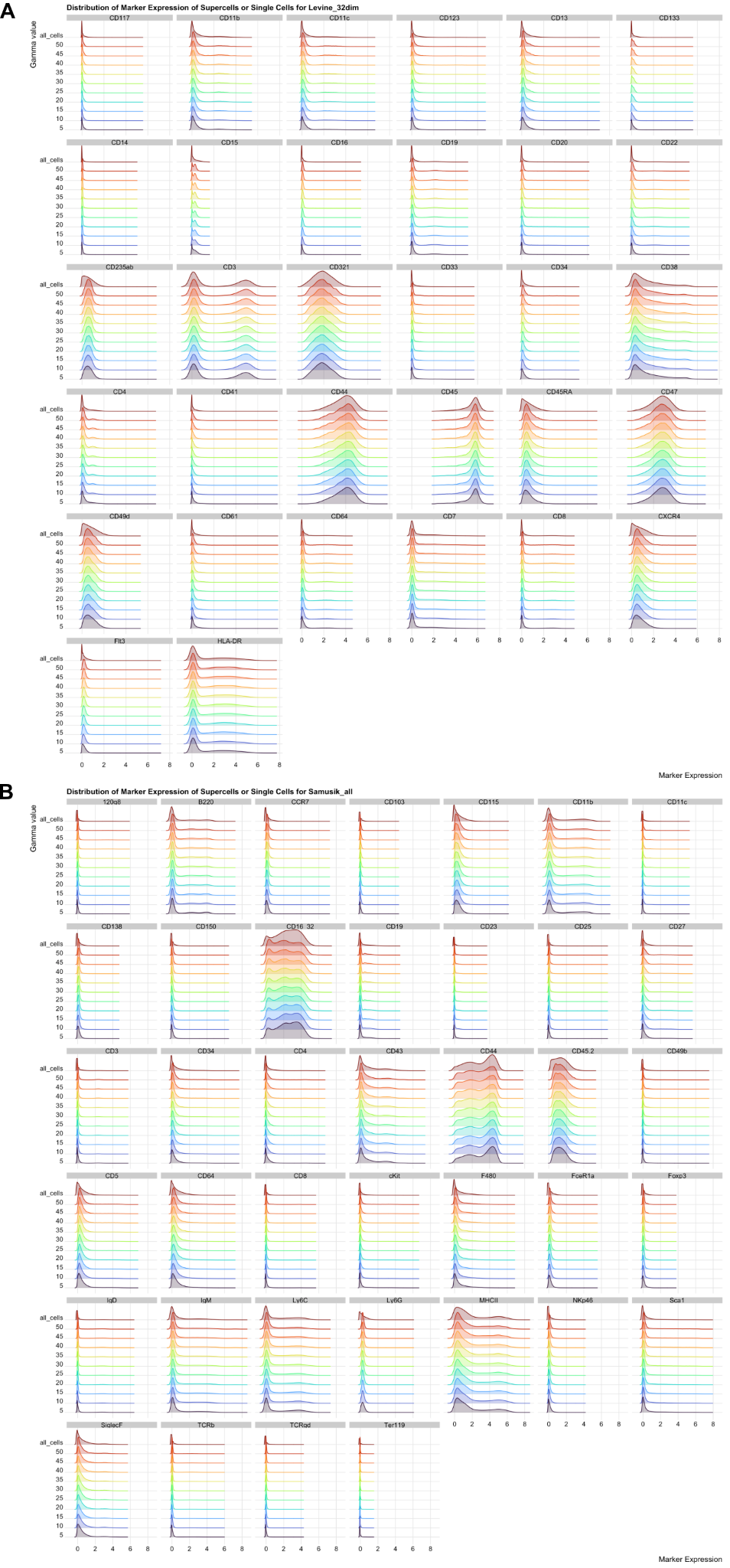


Fig. S5. Distribution of marker expression for supercells generated using gamma values ranging from 5 to 50 (in increments of 5), and for single cells (denoted by the 'all_cells' row label) for Levine_32dim [[7]](https://www.zotero.org/google-docs/?zl46DV) and Samusik_all [[8]](https://www.zotero.org/google-docs/?OrLJUg) datasets.


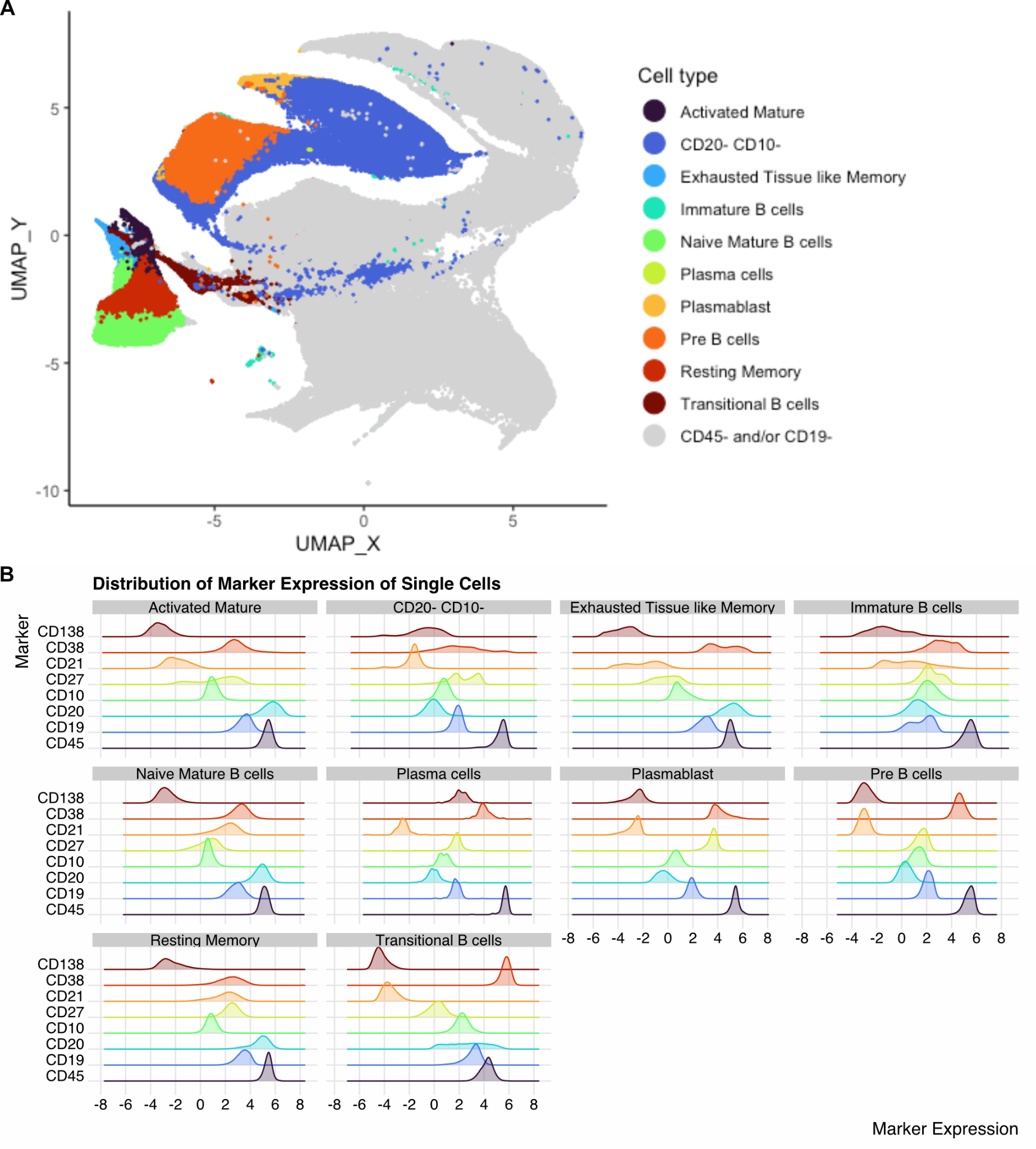


Fig. S6. UMAP plot and marker expression distribution for Oetjen_bcells data [[45]](https://www.zotero.org/google-docs/?HKwwjz). (A) UMAP plot illustrating supercells generated for the Oetjen_bcells dataset. Each point represents a supercell, and coloured according to the cell type it represents. (B) Distribution of marker expression for single cells in the annotated supercells. Single cells were extracted from annotated supercells, and assigned the corresponding supercell’s annotation.


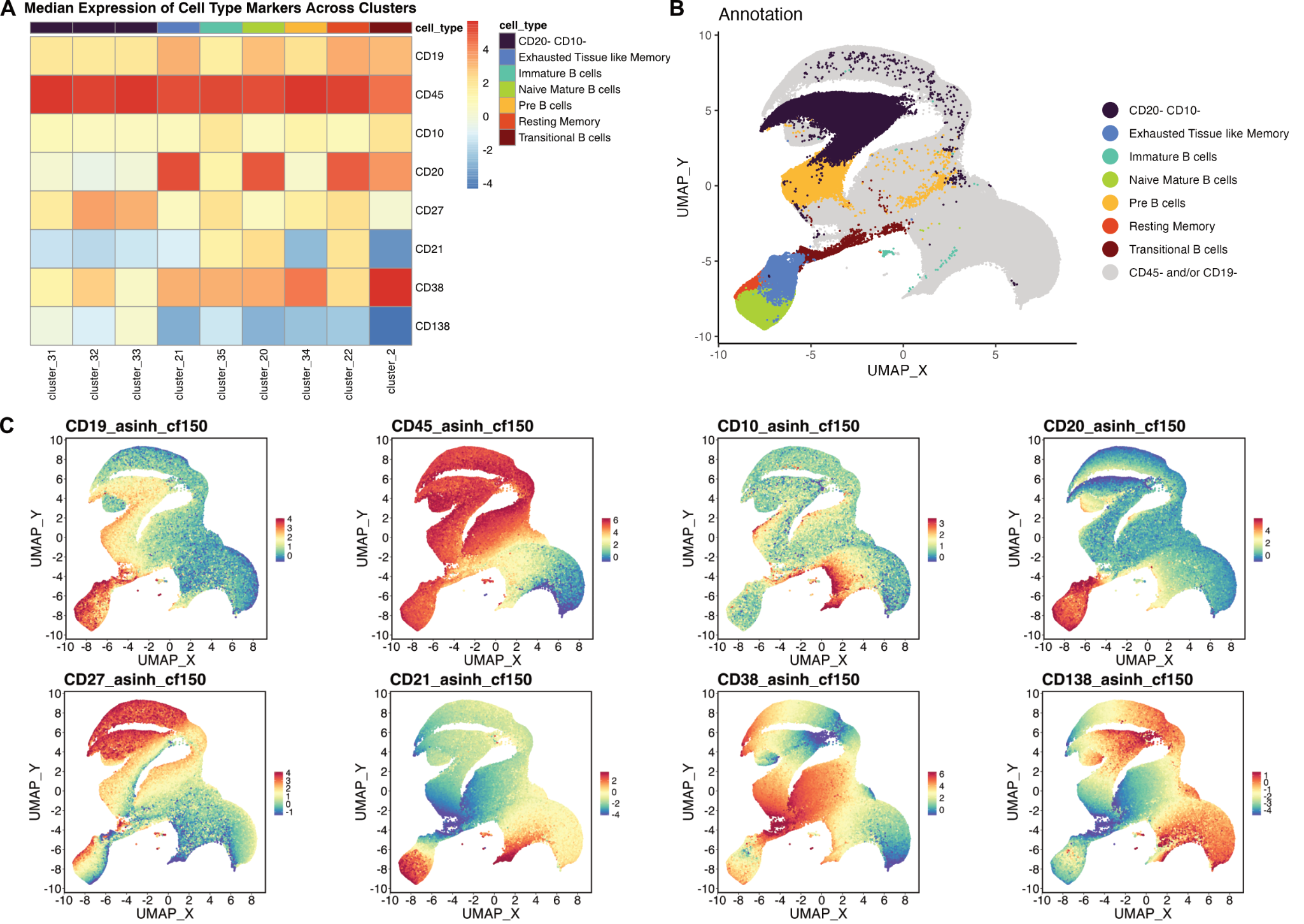


Fig. S7. Heatmap and UMAP plots for randomly subsampled 415,711 cells from the Oetjen_bcells data [[45]](https://www.zotero.org/google-docs/?NiVbTN). (A) Median expression of cell type markers. Only clusters assigned one of the B cell subsets are shown (B-C) UMAP plots illustrating the B cell subsets identified and the expression of the cell type markers used to annotate the cells.


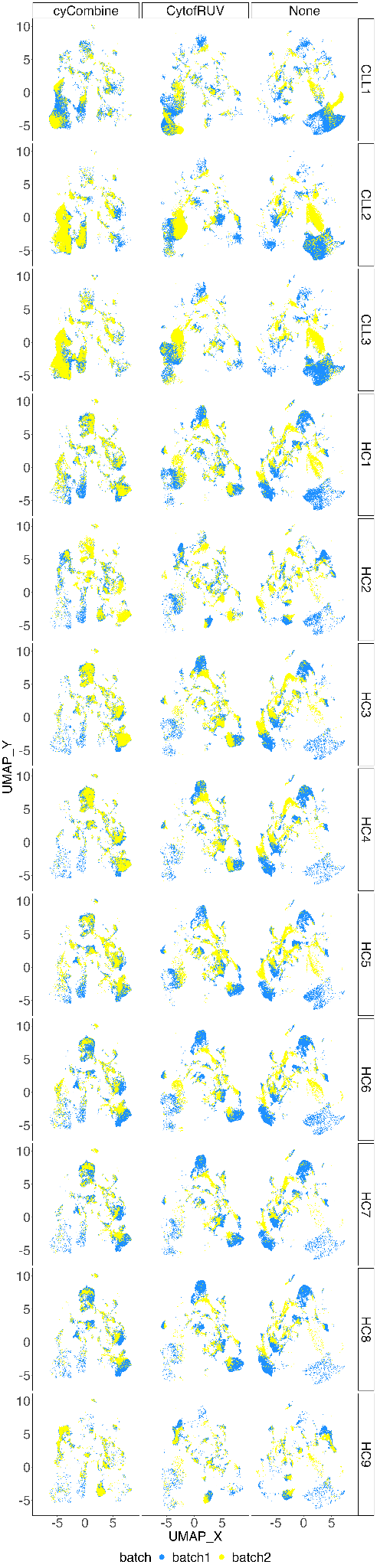


Fig. S8. UMAP plots of supercells for Trussart_cytofruv dataset [[46]](https://www.zotero.org/google-docs/?Q4EtpG). Each row represents the UMAP plots corresponding to a specific paired sample from a patient, with supercells either corrected using CytofRUV or cyCombine, or left uncorrected.


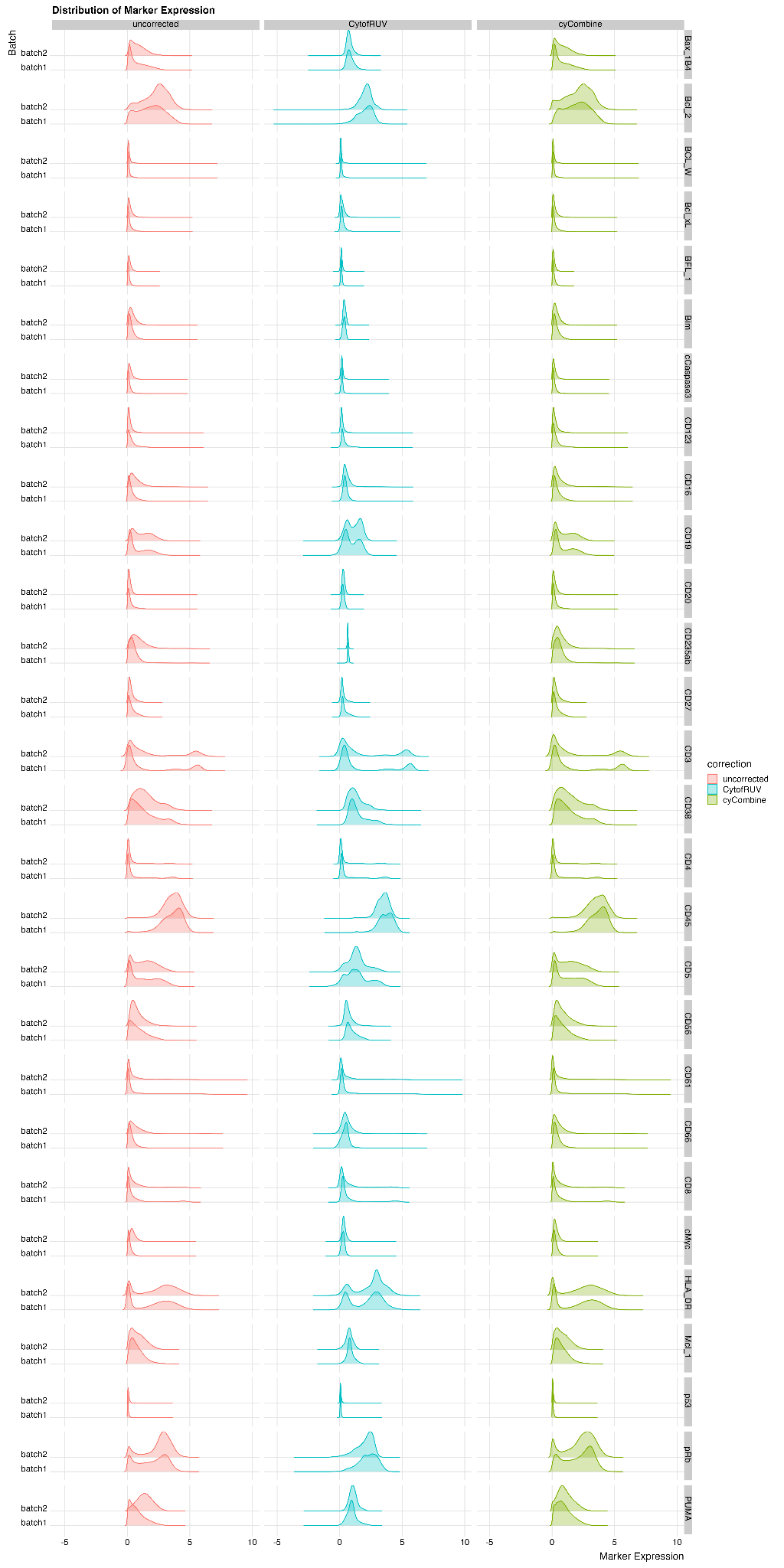


Fig. S9. Distribution of marker expression for uncorrected, CytofRUV-, and cyCombine-corrected supercells for Trussart_cytofRUV [[46]](https://www.zotero.org/google-docs/?476KMk) dataset.


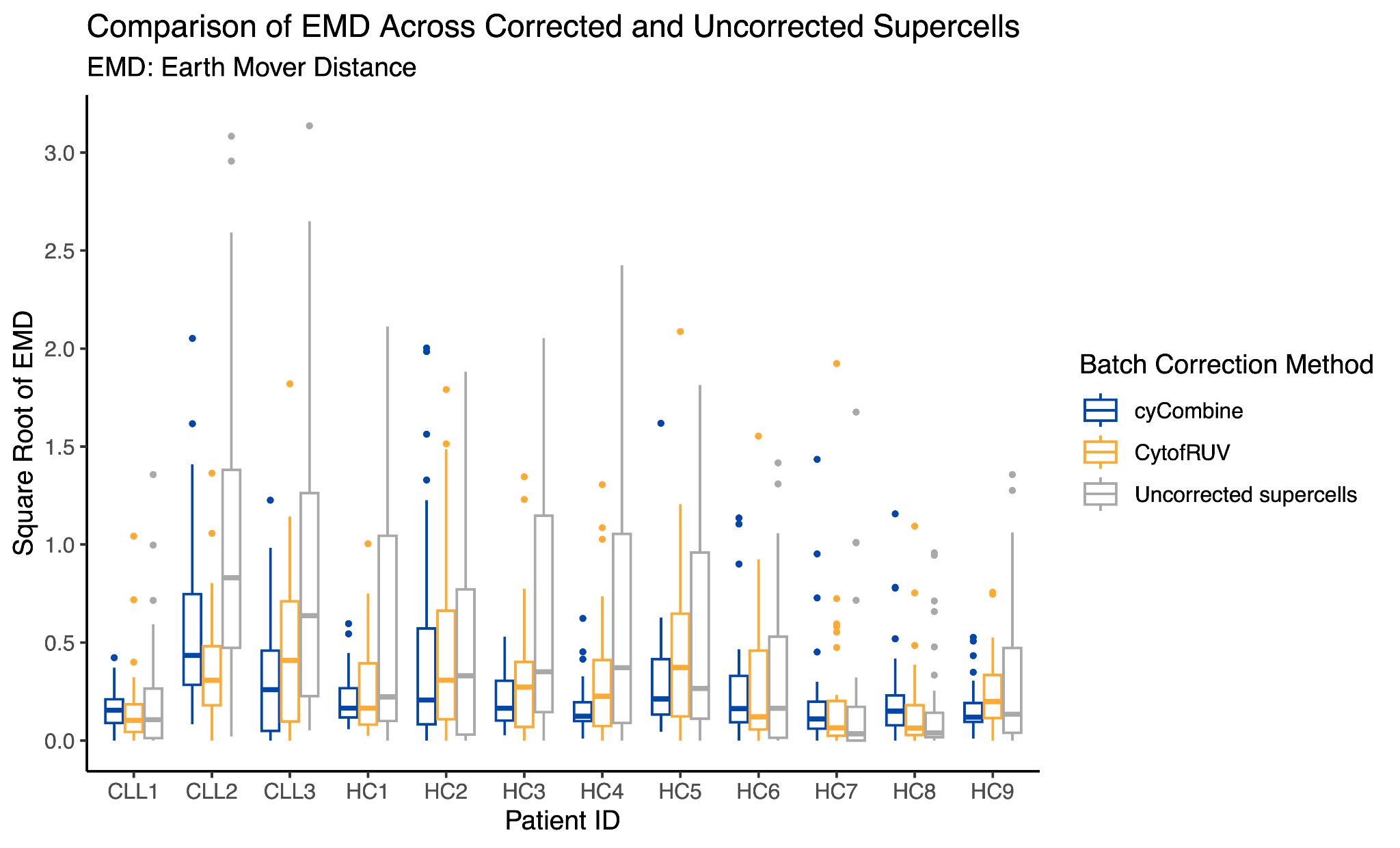


Fig. S10. A comparison of the Earth Mover Distance (EMD) calculated for all markers before and after the application of batch effect correction for Trussart_cytofRUV dataset [[46]](https://www.zotero.org/google-docs/?VyTZXB). EMD values on the y-axis underwent a square-root transformation to facilitate data visualisation and interpretation.
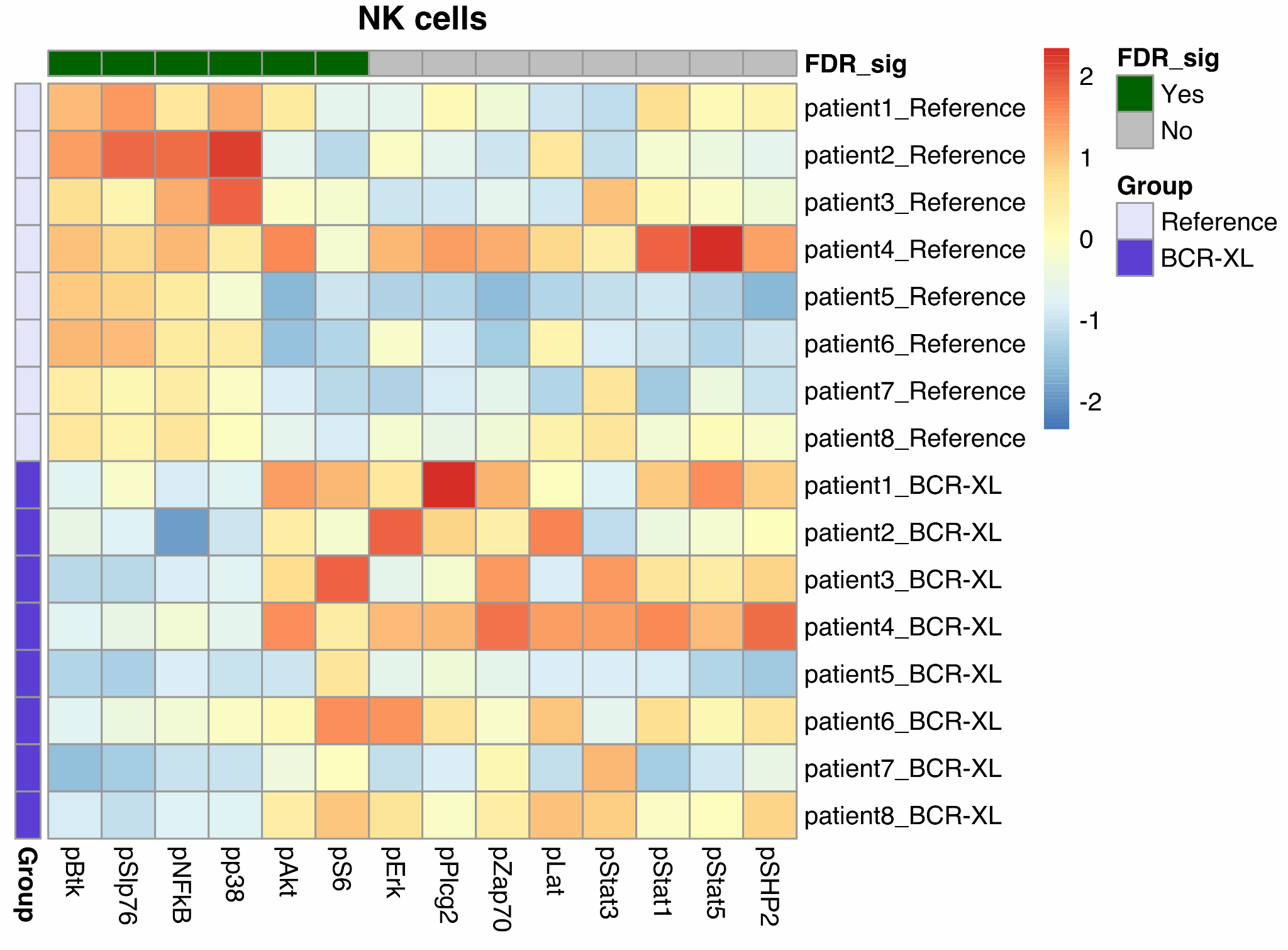


Fig. S11. Differential expression analysis results generated by Limma [[40]](https://www.zotero.org/google-docs/?u4M8y2) for NK cells in the BCR_XL dataset [[49]](https://www.zotero.org/google-docs/?kwZDBH). The heatmap illustrates the scaled and centred median expression of cell state markers, calculated for each sample across the supercells.


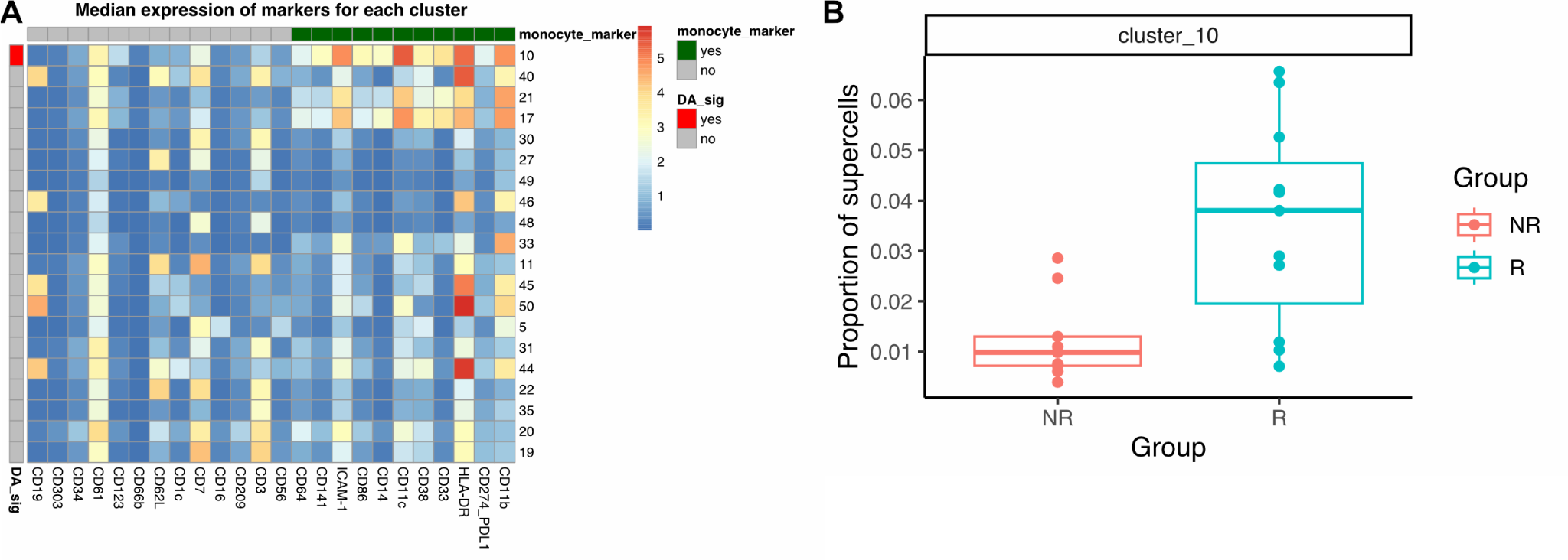


Fig. S12. Differential abundance analysis for the Anti-PD1 dataset [[50]](https://www.zotero.org/google-docs/?YV5C0k). (A) Heatmap depicting the median expression of markers for each cluster calculated across the supercells. Each marker (column) is annotated based on its function for identifying the rare monocyte subset whose abundance is strongly associated with melanoma patients’ responder status to anti-PD-1 immunotherapy (monocyte_marker). Each cluster (row) is annotated based on the statistical significance of their abundance variation as determined by Propeller (DA_sig, FDR <= 0.05). (B) The proportion of supercells for cluster 10 which represents the rare monocyte subset.


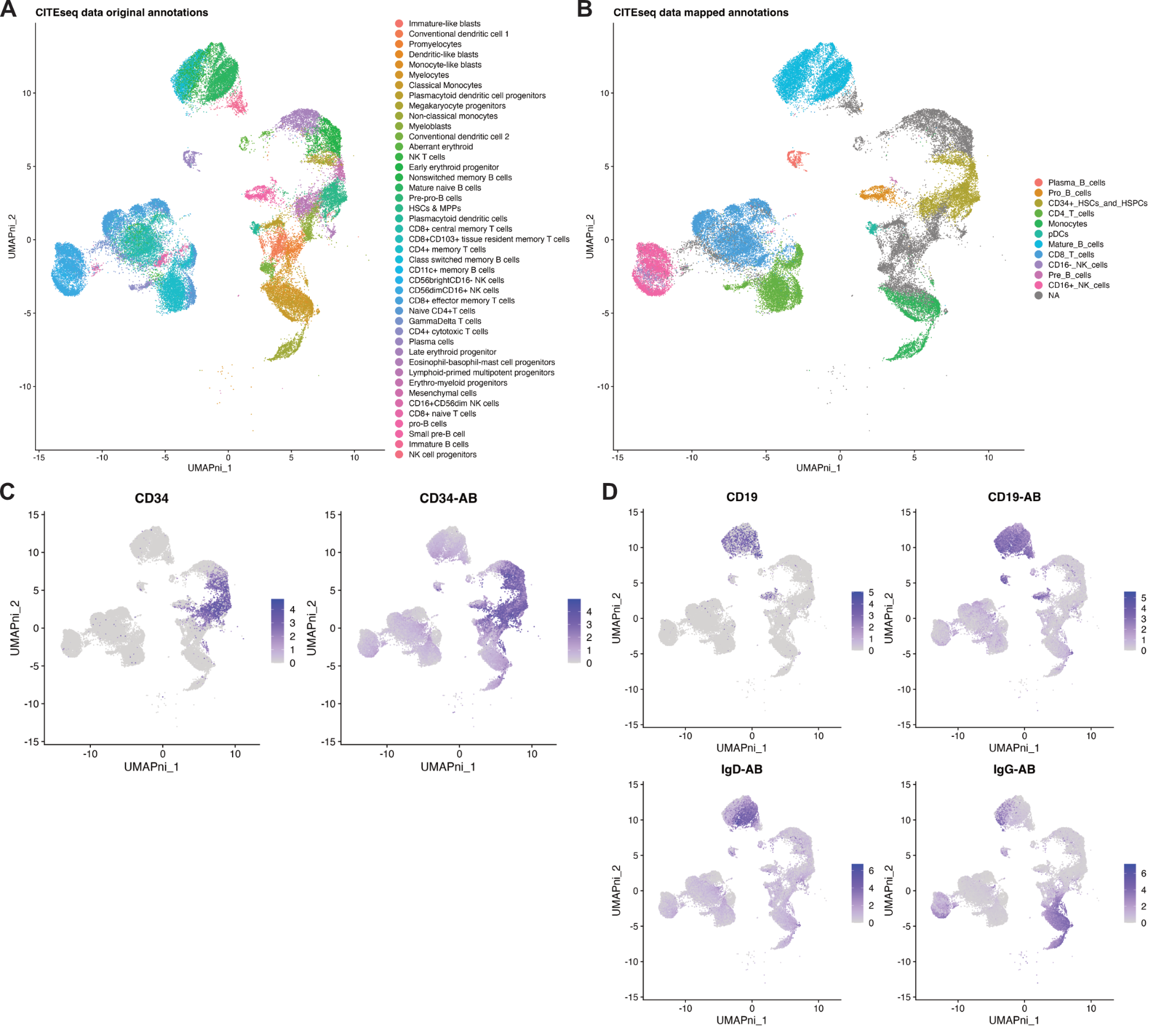


Fig. S13. UMAP plots of the CITEseq data [[52]](https://www.zotero.org/google-docs/?KhikKS) used in label transfer analysis. Plots are coloured by (A) the original cell type label annotations provided by the authors of the data [[52]](https://www.zotero.org/google-docs/?ugdbgR), (B) the mapped cell type labels for annotating the cytometry data used to calculate accuracy and weighted accuracy metrics, (C) CD34 RNA and antibody (CD34-AB) expressions, (D) CD19 RNA, CD19 (CD19-AB), IgD, and IgG antibodies expressions.


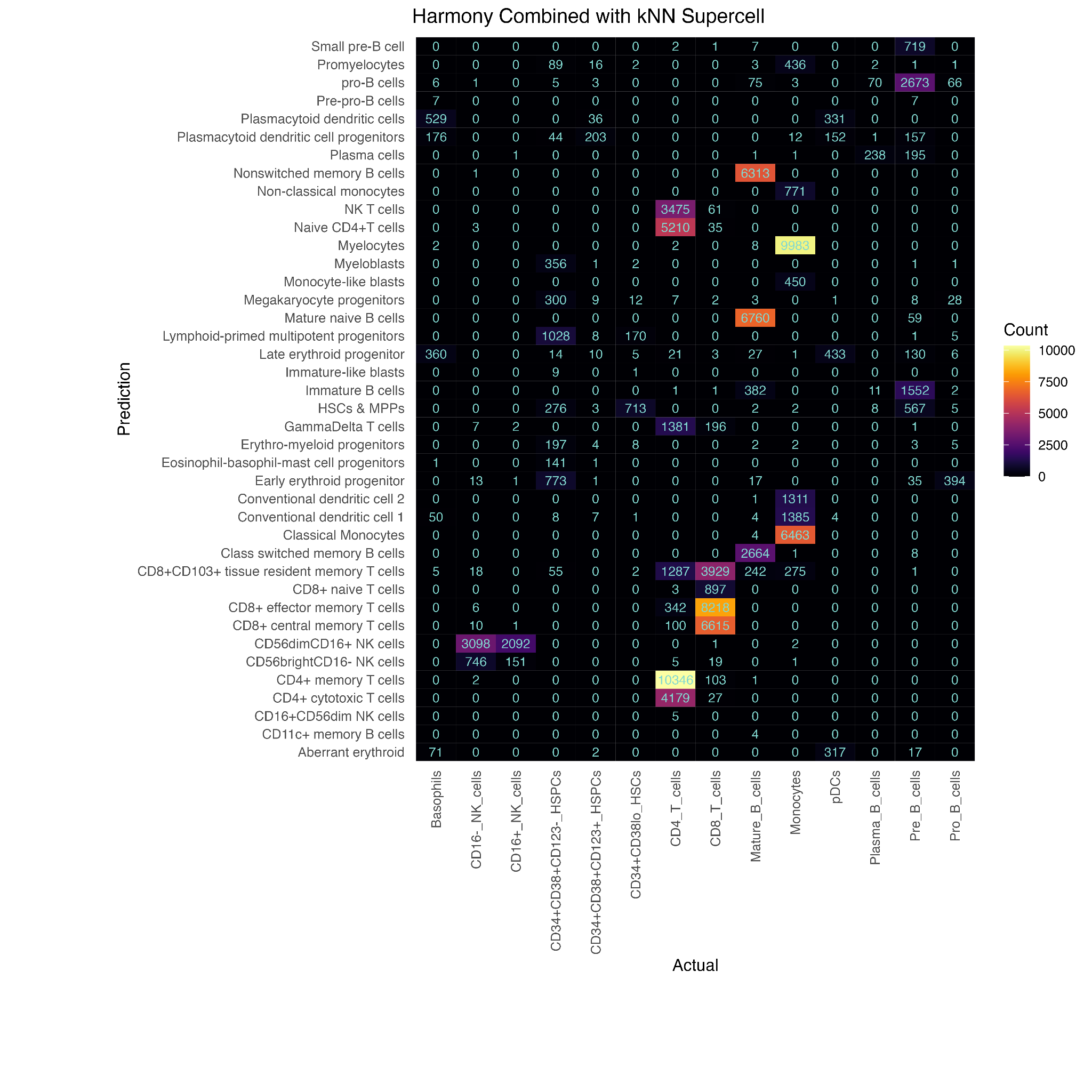
Fig. S14. Performance evaluation of the cell type label transfer workflow, from a CITEseq dataset to supercells generated for the Levine_32dim cytometry data using Harmony [[51]](https://www.zotero.org/google-docs/?SChm5f) combined with a k-Nearest Neighbour (kNN) classifier.


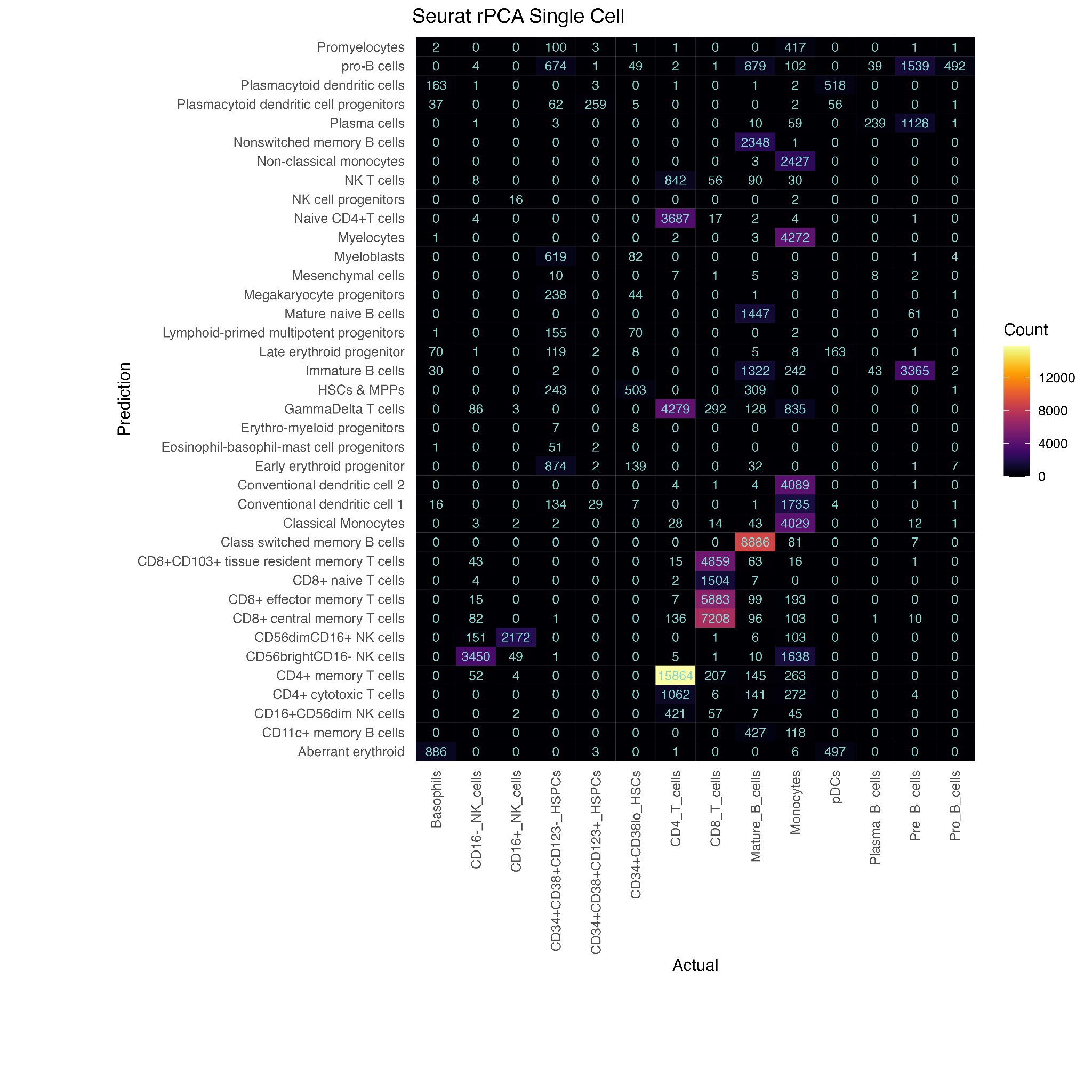
Fig. S15. Performance evaluation of the cell type label transfer workflow, from a CITEseq dataset to the single cells in the Levine_32dim cytometry data, using Seurat rPCA [[16]](https://www.zotero.org/google-docs/?a3XEPd).


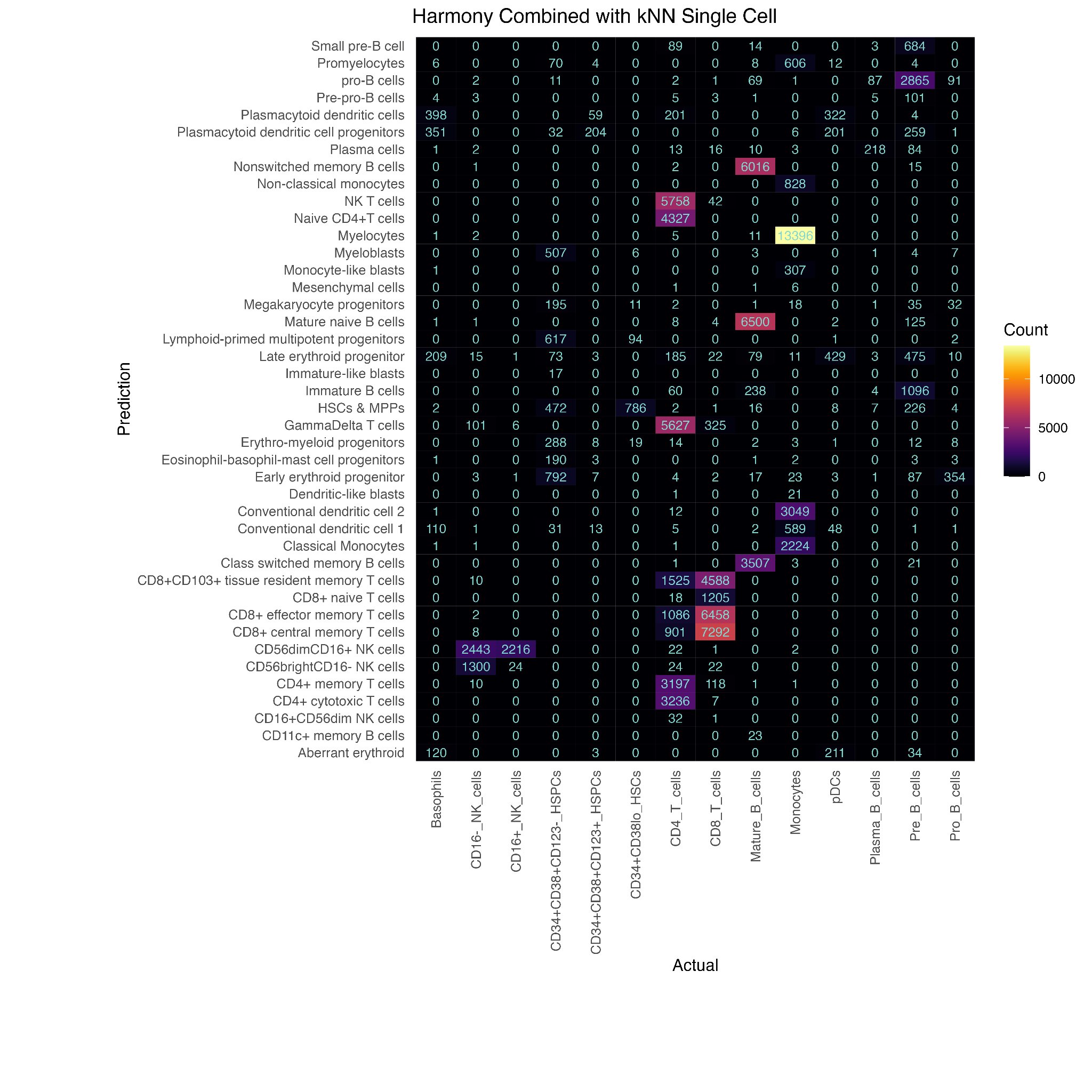
Fig. S16. Performance evaluation of the cell type label transfer workflow, from a CITEseq dataset to single cells in the Levine_32dim cytometry data using Harmony [[51]](https://www.zotero.org/google-docs/?p2QzbS) combined with a k-Nearest Neighbour (kNN) classifier.


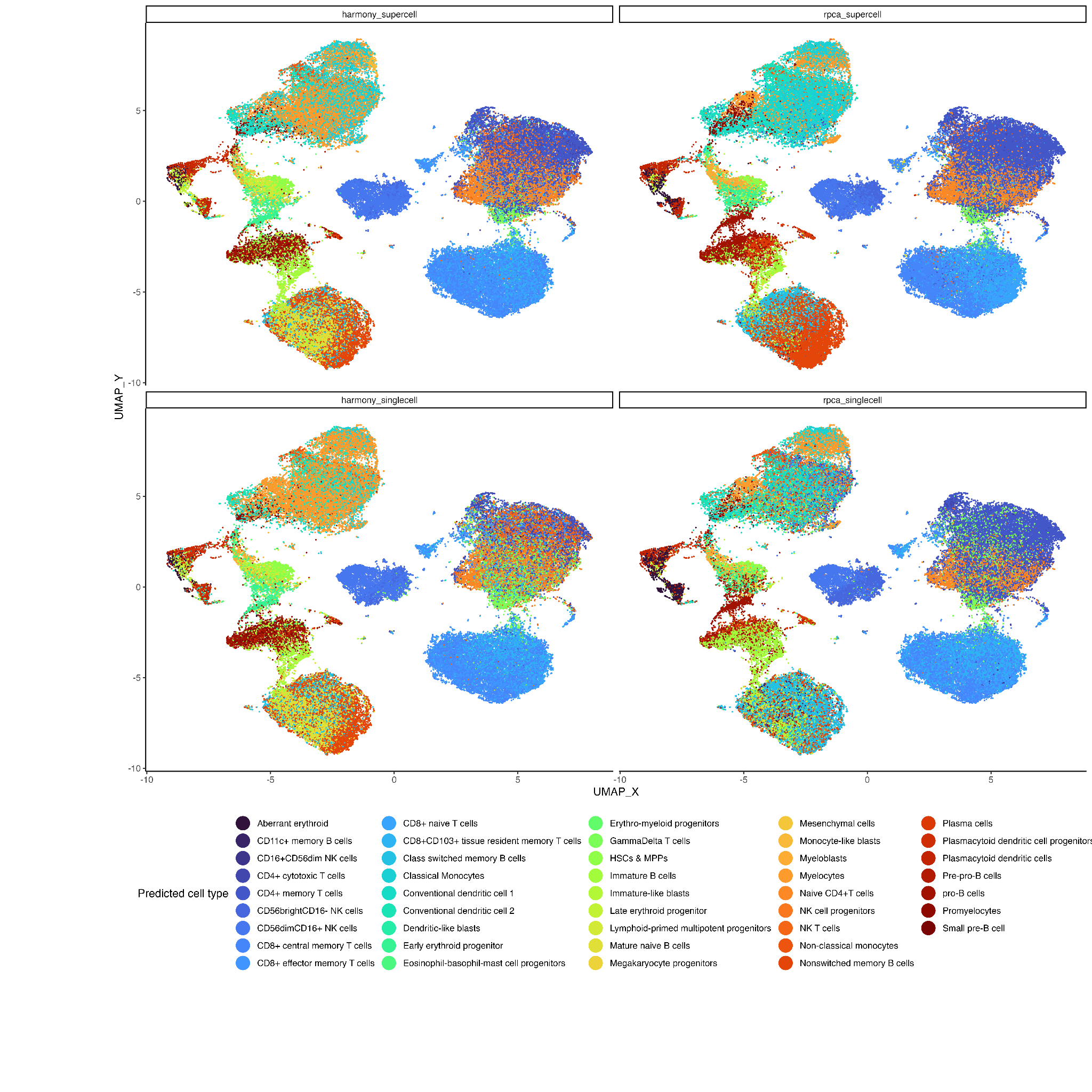


Fig. S17. UMAP plots of single cells for Levine_32dim dataset [[52]](https://www.zotero.org/google-docs/?jqdszk) annotated using either Harmony [[51]](https://www.zotero.org/google-docs/?wi7Jir) combined with a k-Nearest Neighbour (kNN) classifier or Seurat rPCA [[16]](https://www.zotero.org/google-docs/?OYQXyc) applied at either the single cell or the supercell level. Only cells assigned cell type labels using manual gating, i.e., the true label is known, are shown.


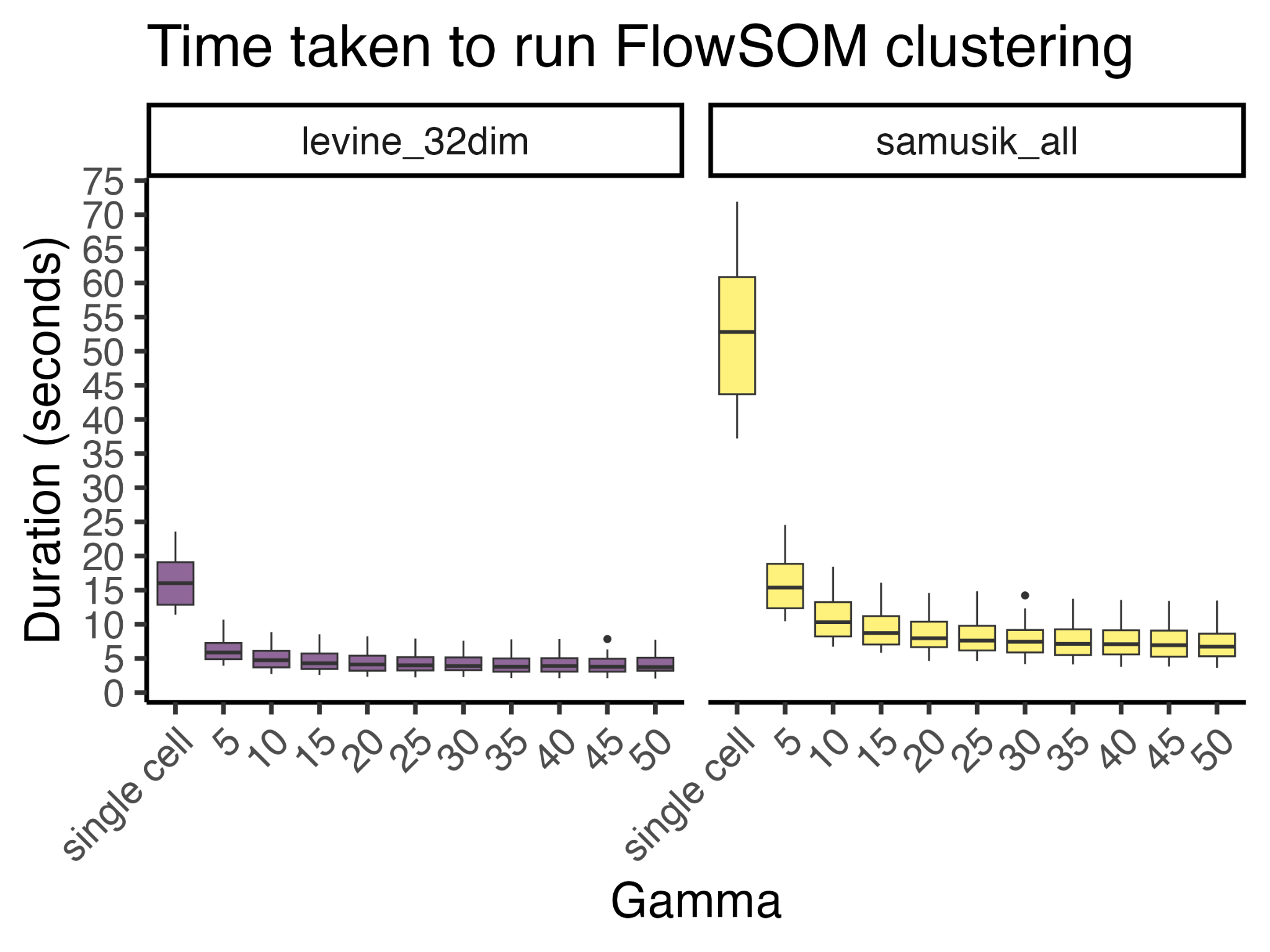


Fig. S18. The runtime of FlowSOM [[6]](https://www.zotero.org/google-docs/?WzXVmz) clustering for Levine_32dim [7] and Samusik_all [8] datasets, measured in seconds


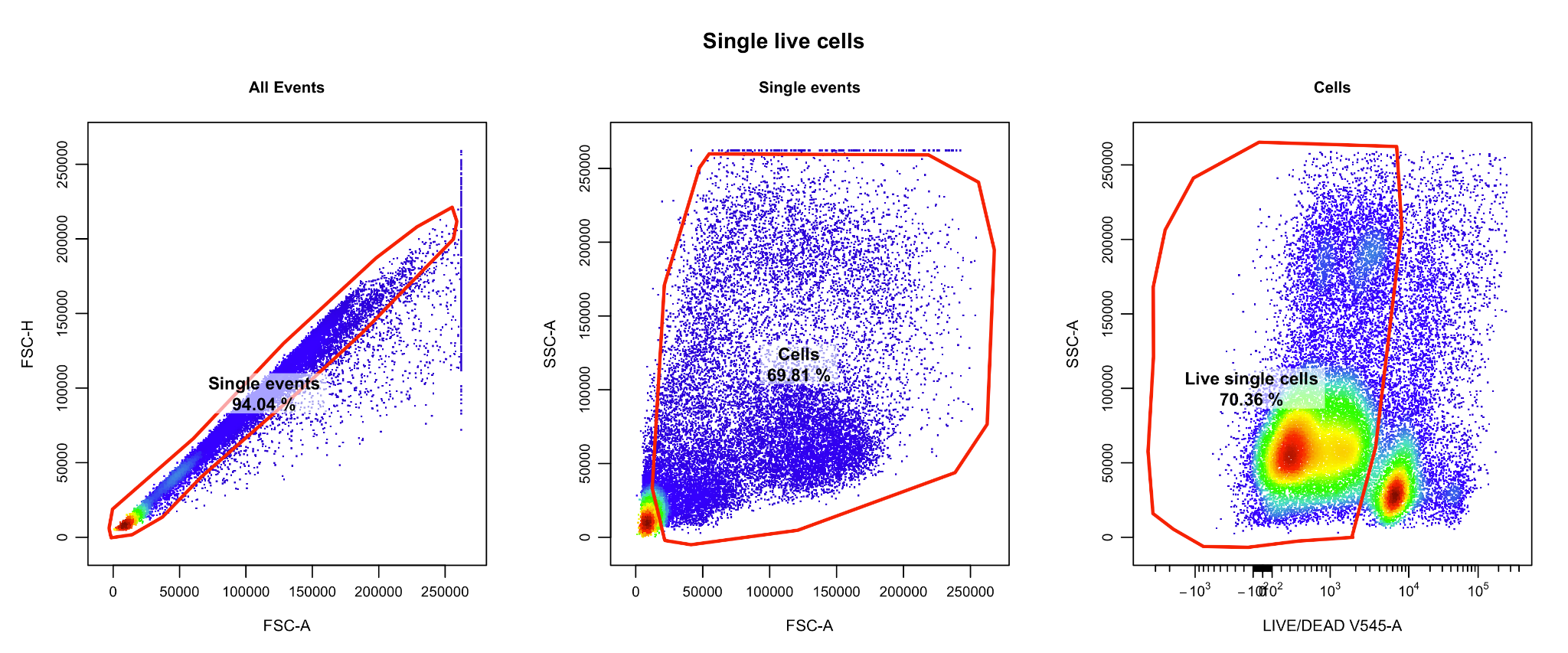


Fig. S19. The manual gating strategy used to isolate single live cells from the Oetjen_bcells [45] flow cytometry dataset.
